# Micro- and nanotechnology in biomedical engineering for cartilage tissue regeneration in osteoarthritis

**DOI:** 10.3762/bjnano.13.31

**Published:** 2022-04-11

**Authors:** Zahra Nabizadeh, Mahmoud Nasrollahzadeh, Hamed Daemi, Mohamadreza Baghaban Eslaminejad, Ali Akbar Shabani, Mehdi Dadashpour, Majid Mirmohammadkhani, Davood Nasrabadi

**Affiliations:** 1 Department of Medical Biotechnology, School of Medicine, Semnan University of Medical Sciences, Semnan, Iran; 2 Biotechnology Research Center, Semnan University of Medical Sciences, Semnan, Iran; 3 Department of Chemistry, Faculty of Science, University of Qom, Qom 37185-359, Iran; 4 Department of Cell Engineering, Cell Science Research Center, Royan Institute for Stem Cell Biology and Technology, ACECR, Tehran, Iran; 5 Department of Stem Cell and Developmental Biology, Cell Science Research Center, Royan Institute for Stem Cell Biology and Technology, ACECR, Tehran, Iran; 6 Department of Epidemiology and Biostatistics, Faculty of Medicine, Semnan University of Medical Sciences, Semnan, Iran

**Keywords:** biological cues, cartilage regeneration, micro/nanotopographical cues, nanotechnology, osteoarthritis, regenerative medicine

## Abstract

Osteoarthritis, which typically arises from aging, traumatic injury, or obesity, is the most common form of arthritis, which usually leads to malfunction of the joints and requires medical interventions due to the poor self-healing capacity of articular cartilage. However, currently used medical treatment modalities have reported, at least in part, disappointing and frustrating results for patients with osteoarthritis. Recent progress in the design and fabrication of tissue-engineered microscale/nanoscale platforms, which arises from the convergence of stem cell research and nanotechnology methods, has shown promising results in the administration of new and efficient options for treating osteochondral lesions. This paper presents an overview of the recent advances in osteochondral tissue engineering resulting from the application of micro- and nanotechnology approaches in the structure of biomaterials, including biological and microscale/nanoscale topographical cues, microspheres, nanoparticles, nanofibers, and nanotubes.

## Review

### 1 Introduction

Osteoarthritis (OA) is a widespread degenerative disease of articular cartilage, which causes severe disability, deformity, and pain in the joints of patients [1]. The most common treatment for lesions of articular cartilage is based on medical treatment, but joint replacement surgery has been recommended for patients with severe cartilage destructions. However, these therapies are not very effective in the long term and impose more cost on patients with OA [2]. Therefore, it is necessary to find novel and alternative approaches for the treatment of this chronic and progressive disease.

Over the past years, massive advances in stem cell technology have been made and offered hope for the treatment of degenerative diseases [3]. Articular cartilage defects were one of the first potential candidates for tissue engineering (TE) applications due to their anural and avascular integrity. Many efforts have been devoted to developing scaffolds with similar structures and functions to native cartilage. Nevertheless, despite the progresses made in biomaterial synthesis, scaffold fabrication, and development of growth factor delivery systems, TE has been unable to address OA disease using the routine methods.

Recently, more attention has been paid to the involvement of micro-/nanotechnological possibilities in the process of scaffold design and fabrication [4]. Since a lot of cellular mechanisms are controlled by environmental stimuli (cell signaling molecules, extracellular matrix, and bioactive macromolecules) on the nanoscale, the recognition and application of such stimuli pave the way for the application of new and promising sources for re-engineering of biomaterials.

**Figure 1:**
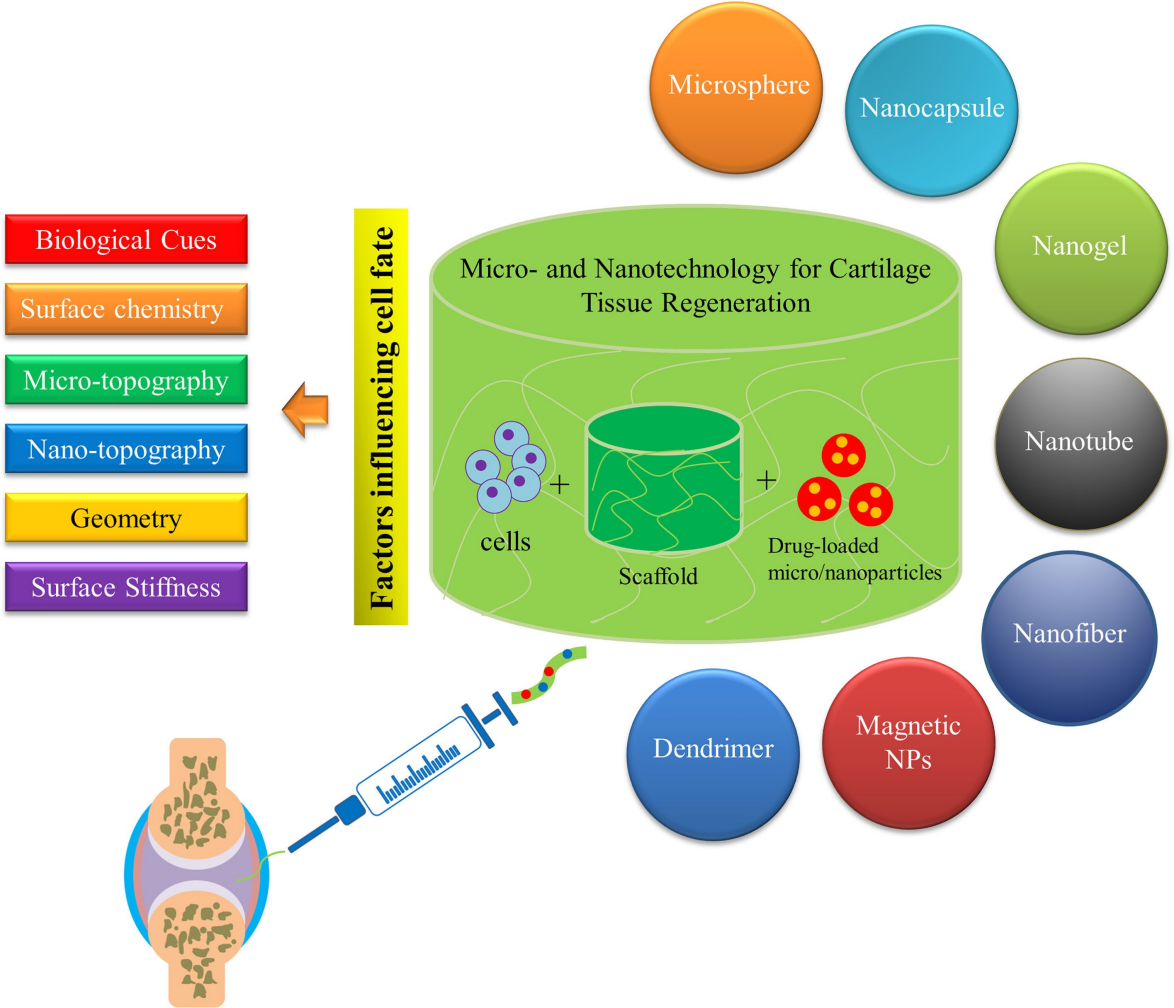
A schematic representation of the issues covered in this review.

In this paper, the native structure of articular cartilage is briefly described due to the importance of understanding its complex structure before designing a suitable construct. Afterward, recent advances in osteochondral tissue engineering resulting from the application of microspheres, nanoparticles, nanofibers, and nanotubes in the structure of biomaterials will be covered (Figure 1). Finally, the role of various cues such as biological cues, microscale/nanoscale topographical cues, and surface stiffness in osteochondral tissue differentiation and engineering is discussed with examples from recent studies that highlight their importance in tissue engineering applications.

### 2 Articular cartilage biology

**Figure 2:**
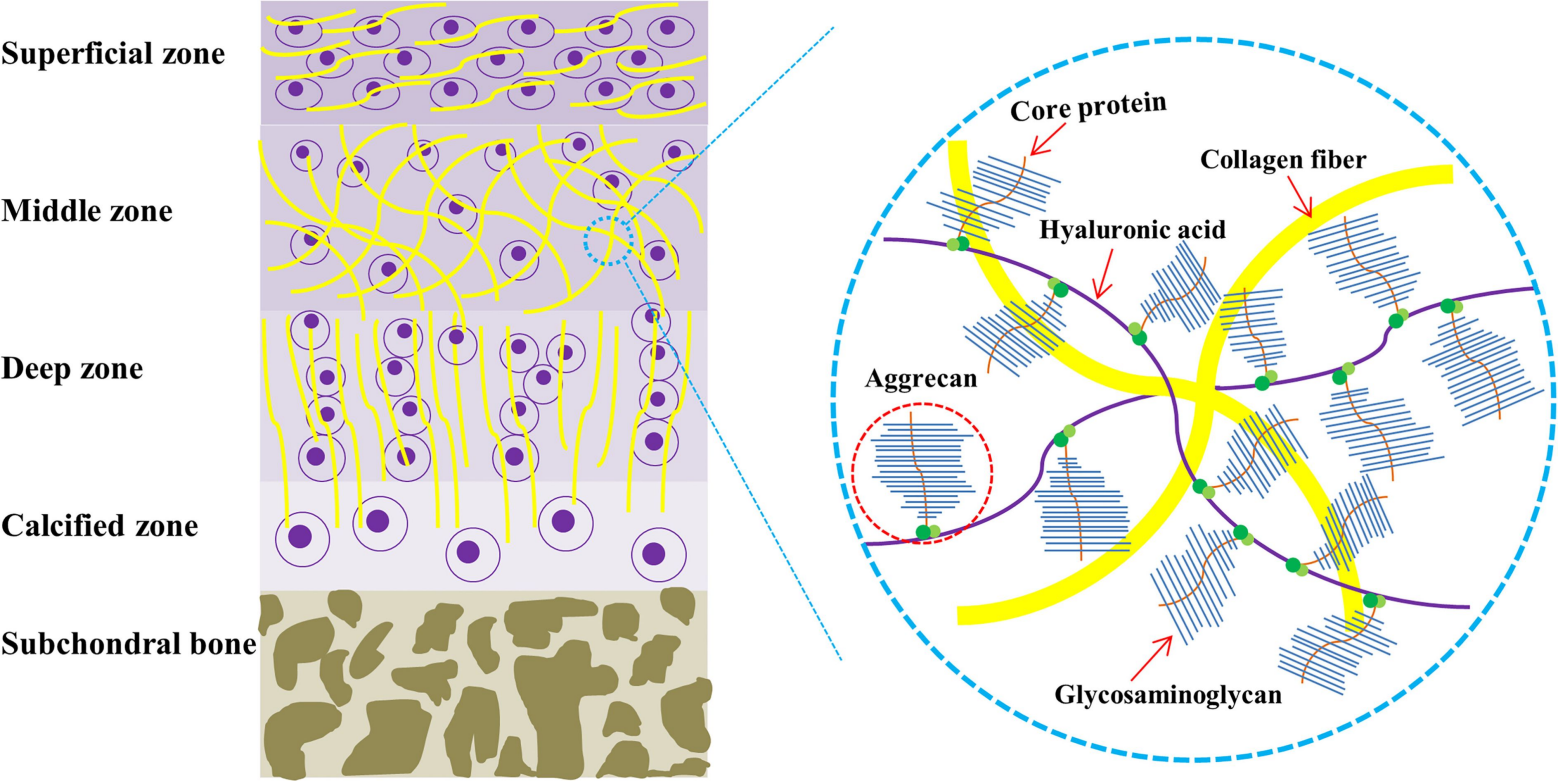
Simplified graphical representation of a cross section of articular cartilage and its associated molecular components. Articular cartilage is normally divided into four distinct regions (the superficial, middle, deep, and calcified zones) visually characterized by the orientation of the collagen fibrils and chondrocyte morphology.

The composition and complex structure of articular cartilage need to be understood before developing a mimicking construct. Articular cartilage, which is characterized by its extensive extracellular matrix (ECM), is a highly specialized connective tissue [5]. Hyaline cartilage is a semitransparent tissue located in the articular cartilage of all joints where it provides a low-friction, wear resistant surface for joint motion [6–7]. It is an avascular, aneural, alymphatic, and hypocellular tissue consisting of a single cell type (chondrocyte) dispersed in a dense matrix [6]. Chondrocytes, which constitute only about 5% of the wet weight of the articular cartilage, are responsible for the synthesis and maintenance of hyaline cartilage matrix [8]. Chondrocytes are embedded in a dense ECM composed mainly of water, glycosaminoglycans (GAGs), and collagen fibers. Collagen comprises 60–85% of adult articular cartilage and has a fibrous structure, which extensively contributes to the mechanical properties of cartilage [7,9]. Collagen type II is believed to account for 75% of fetal collagen. This amount increases to 90% in mature adults. Collagen type II is found in articular cartilage and the eyes and is considered an indicator of hyaline cartilage [7]. Collagen synthesis is slow and plays a critical role in cartilage functionality and homeostasis. Therefore, any degradation would result in the damage and failure of cartilage to withstand mechanical loads. Hyaluronic acid (HA) is an unbranched polysaccharide with a high molecular weight, which plays a major role in gathering and aggregating proteoglycans due to the high affinity of aggrecan for it [10] (Figure 2). Hence, articular cartilage is a negatively charged complex of collagen fibers and HA conjugated proteoglycans highly organized into a stratified structure, which can withstand mechanical stresses [6].

The hierarchical structure of articular cartilage from its surface to the subchondral bone shows a depth-dependent transition and anisotropic integrity composed of four distinct zones; namely, superficial, middle, deep, and calcified cartilage [11–12] (Figure 2). The arrangement, composition, and content of the cartilage matrix vary in each layer [5]. The superficial zone contains a relatively dense number of flattened and randomly dispersed chondrocytes with collagen fibrils arranged parallel to the articular surface [13]. This zone is responsible for the highest tensile properties of articular cartilage and supports deeper layers from shear stresses [5]. The middle or transitional zone constitutes the thickest portion of articular cartilage (40–60%) and has fewer chondrocytes with a more rounded morphology [6]. In this layer, the collagen fibrils are arranged randomly and obliquely and the cells synthesize relatively greater amounts of the proteoglycans [14]. The deep zone consists of the lowest cell density, highest aggrecan content, largest diameter collagen fibrils, and least amount of collagen [12]. In this layer, the chondrocytes are typically arranged in columnar clusters parallel to the collagen fibrils and vertical to the articular surface. Meanwhile, this zone provides the greatest resistance to compressive forces [6]. The calcified cartilage zone, which is presumed to be an interface layer between the upper cartilage layers and the rigid subchondral bone, contains chondrocytes, which usually express hypertrophic markers such as type X collagen and alkaline phosphatase (ALP). Mineralization occurs after the tidemark, which is a thin wavy layer between the deep zone and calcified layer [15].

Overall, the complexity of the hierarchical structure and considerable differences visible in the gene expression profile of articular cartilage pose important challenges for TE.

### 3 Cartilage TE

OA is a prevalent joint disease characterized by mechanical instability of the joints, cartilage destruction, subchondral bone thickening, inflammation of the synovium, osteophyte formation, degeneration of ligaments, and capsule hypertrophy [1]. Although joint replacement surgery is recommended in patients with severe OA, techniques such as autologous chondrocyte implantation (ACI) [16] to treat the damaged cartilage layer present an alternative approach for restoration of the biological and mechanical activities of the joint. However, despite promising results, autologous implantation is limited due to the finite availability of cells, particularly for the elderly, and the possibility of dedifferentiation during the in vitro expansion of biopsy-isolated chondrocytes [17]. These limitations to treat osteochondral defects can be overcome by the versatile and efficient methods developed by TE technologies and will be discussed in detail.

#### 3.1 Development of biomaterials using micro and nanostructures for cartilage TE

Since Vacanti et al. reported the application of bioabsorbable artificial polymers as matrices for cell transplantation in the late 1980s [18], there have been numerous developments in the design and fabrication of bioinspired and smart biomaterials with improved potential of TE as a regenerative medicine approach. The development of hydrogel-based scaffolds for regenerative medicine purposes is the subject of many studies [19] in which natural polymers, synthetic polymers, or their combination were used to provide a biomimetic microenvironment, which not only includes biological cues, but also provides the desired mechanical properties. The progress in materials science has revealed different characteristics and kinds of behavior of compounds on submicrometer and nanometer scales and resulted in the development of new methods such as electrospinning, self-assembly, phase separation, nano-imprinting, and photolithography for the generation of new biomaterials with improved properties. Micro- and nanostructures including microspheres, NPs, nanofibers, nanotubes, and nanofilms have been designed to construct new scaffolds and or incorporated into the hydrogel network to provide a controlled release or enhanced mechanical characteristics. Many of these substructures are widely used for engineering cartilage tissue structures.

**3.1.1 Application of microspheres in chondrogenic differentiation.** Microspheres are used in drug delivery systems because of their spatiotemporally controlled release capabilities (see Table 2 below). They are small spherical particles from organic or inorganic compounds with diameters from 1 to 1000 μm prepared by physicochemical methods [20]. A microsphere-based controlled release strategy is extensively used in scaffold fabrication because of the remarkable ability of microparticles to serve as carriers for delivery of drugs, bioactive molecules, and growth factors in the microsphere-incorporated scaffolds. Microspheres can be independently assembled and used as building blocks for microsphere-based scaffold structures to provide the desired mechanical properties and improve the capability to control release [21]. Designing a properly controlled release system in the context of cartilage regeneration meets the specific challenges, which limit the local delivery of bioactive molecules. In vitro chondrogenesis relies on the gradual addition of growth factors to the culture medium, which is impractical in vivo. The development of a series of scaffolds equipped with controlled release systems for chondrogenic differentiation is a major concern of recent investigations. Ansboro et al. have developed a hyaluronan-based chondromimetic microsphere system to deliver transforming growth factor beta 3 (TGF-β3) growth factor for in situ chondrogenic differentiation [22]. Using this method, they observed enhanced chondrogenic differentiation of human mesenchymal stem cells (hMSCs) aggregated in micromass culture systems including microspheres. The use of a microsphere delivery system for controlled release obviates the need for the intermittent addition of growth factors and enables in situ spatial differentiation of MSCs to repair osteochondral defects [23]. It has been reported that microsphere-based structures could be efficiently used for gradient formation [24] and dual growth factor delivery [25].

Microspheres can be incorporated throughout the scaffold by mixing them into the scaffold structure. Once incorporated, they provide new properties and possibly improve physical characteristics. In a study of in vitro and in vivo MSC chondrogenesis [26], the researchers encapsulated TGF-β3 in alginate microspheres coated with biofilm and subsequently incorporated them into an HA hydrogel. The structures showed superior mechanical properties and longer release of growth factor for more than six days compared to both a hydrogel scaffold loaded directly with TGF-β3 and one with non-coated microspheres. Incorporation of the TGF-β3 loaded microsphere into an MSC seeded hydrogel not only improved the mechanical properties of the structures and expression of chondrogenic markers in vitro, but also enhanced cartilage matrix deposition when subcutaneously implanted in nude mice [26].

Growth factor supplementation is an important prerequisite for the in vivo chondrogenesis of MSCs and cartilage regeneration, which should be performed over a period of several days or weeks. However, a few of the proposed methods achieved the prolonged sustained release of bioactive molecules. For example, low molecular weight polyethyleneimine precoated poly(lactide-*co*-glycolide) (PLGA) microspheres coated with TGF-β3 loaded heparin/poly(ʟ-lysine) NPs showed extended growth factor release for 28 days and were found to be excellent structures for cell adhesion and chondrogenic differentiation [27].

Apart from serving as cell adhesion platforms and growth factor supplements, microsphere structures can be independently used as carriers for the delivery of drugs and bioactive molecules to repair cartilage defects. The sustained release of PTH (1-34) from PLGA microspheres improved papain-induced defects in a rat model of OA and retained the bioactivity of the released peptide up to 19 days [28]. In another study, researchers developed PLGA-based microspheres to control the release of rapamycin in the joint [29]. The microspheres provided sustained and controlled release of rapamycin for several weeks, retained drug potency, and prevented OA-like changes in chondrocytes cultured under genomic and oxidative stress conditions. Furthermore, this formulation had a long residence time (>19 days) in the murine joint after intra-articular injection [29]. In addition, microspheres with a higher surface-to-volume ratio can provide a three-dimensional (3D) environment for cell anchorage and growth, thus shortening the expansion process [30–32]. In this regard, Sulaiman et al. explored the 3D culture of MSCs on gelatin microspheres (GMs) in a dynamic culture system for the fast generation of a higher amount of MSCs and the improvement of their chondrogenic differentiation [31]. The results showed that MSCs cultured on GMs in a dynamic culture displayed a faster proliferation rate and higher stemness properties compared with those in two dimensional (2D) and 3D static culture systems [31]. Moreover, culturing MSCs on GMs in a dynamic system with the chondrogenic induction medium (CIM) enhanced their in vitro chondrogenesis compared to a static culture system [31]. Therefore, the combination of the 3D culture system (GM), CIM, and dynamic culture would be an ideal design for MSC based therapies for articular cartilage defects.

**3.1.2 Nanomaterial-assisted cartilage TE.** Nanomaterials are defined by the National Nanotechnology Initiative as manufactured or natural materials that have at least one dimension between 1 and 100 nm [33–46]. Nanomaterials are classified as metals, polymers, or ceramics according to their structure and nature. They are constructed as NPs, nanofibers, nanocrystals, nanotubes, and nanofilms by high-tech methods of photolithography, electrospinning, nanoimprinting, and phase separation. Due to the hierarchical structure of articular cartilage ECM, there is considerable enthusiasm regarding the use of biomimetic nanocomposites to imitate pseudostratified features of the ECM to develop bioinspired scaffolds [47–49].

**3.1.2.1 Nanoparticles (NPs).** In recent years, NPs have been increasingly used in regenerative medicine (Table 1) and other medical areas. NPs have been successfully developed for drug delivery [50], in vitro diagnosis [51], in vivo imaging [52], and TE purposes. Various NPs can be prepared in the form of liposomes, nanocapsules, micelles, dendrimers, and nanospheres based on their composition and method of preparation. Basically, NPs are designed to function as carriers for bioactive molecules and to provide protection for these molecules from physiological degradation. NPs are expected to control the release profile of the incorporated agent, decrease the severity of the side effects of these drugs, and maximize the effect of the bioactive agent through its safe delivery to target cells. The incorporation of NPs loaded with bioactive agents in hydrogels has a tremendous impact on the properties and functionality of tissue-engineered scaffolds. In addition to providing sustained release properties, which is a major challenge in TE programs, the incorporation of NPs can improve the mechanical properties and the simultaneous or sequential delivery of bioactive molecules. In this regard, an injectable gellan xanthan hydrogel containing chitosan NPs has been prepared as a scaffold for the delivery of multiple growth factors to regenerate bone [53]. The results of this study showed that the entrapment of the bone morphogenetic protein 7 (BMP7) and basic fibroblast growth factor (BFGF) within chitosan NPs inhibited the initial burst release and allowed for the controlled release of growth factors and therefore, improved the osteogenic differentiation of stem cells [53]. In another study, silk fibroin/poly(ethylene glycol) dimethacrylate (PEGDMA) hydrogels containing PLGA nanoparticles were used for the simultaneous delivery of bFGF and TGF-β1 to regenerate articular cartilage tissue [54]. The results showed that the simultaneous release of bFGF and TGFß1 improved the viability and proliferation of dental pulp stem cells (DPSCs) as well as chondrogenesis compared with the delivery of a single growth factor [54]. In addition, researchers utilized NPs composed of PLGA and poly(*N*-isopropylacrylamide) (PNIPAM) for the controlled release of insulin-like growth factor I (IGF-I) and TGF-β1 [55]. The results showed that the combination of both growth factors in the loaded system yielded better results in terms of chondrogenic differentiation of MSCs compared to systems with no or only one growth factor [55]. In addition, the spatiotemporal delivery of multiple growth factors using separate loading systems may provide desirable kinetics for the sequential controlled release of growth factors. In line with this, a two-growth-factor system consisting of TGF-β2-loaded NPs encapsulated in a BMP-7-loaded alginate hydrogel enabled a slower release of TGF-β2 and a faster release of BMP-7 by approximately 30% and 80%, respectively, at the end of day 21 [56]. The results showed that the spatiotemporal compartmentalization of growth factors in different delivery systems was responsible for the individual and sequential release and might prevent aggregation of the growth factors.

**Table 1:** Summary of the recent studies on microscale/nanoscale materials used for the fabrication of cartilage tissue engineering (TE) structures.

Microscale/ nanoscale material type	Microscale/ nanoscale material application	Base material	Scaffold used	Cell type	In vivo	Major result	Ref.

Microspheres	The increase in the proliferation rate of MSCs and their chondrogenic differentiation.	Gelatin	Gelatin microsphere	Human BMSCs	—	Culturing MSCs on gelatin microspheres accelerated proliferation rate and preserved stemness properties as well as enhanced chondrogenesis	[31]
Microspheres	Delivery of rapamycin	PLGA	—	Human chondrocytes	Mice model	Provided sustained and controlled release of rapamycin for several weeks and prevented OA-like changes in chondrocytes under genomic and oxidative stress conditions	[29]
Microspheres	Verteporfin delivery	Chitosan	Collagen I-coated culture dish and PDMS substrates	Human chondrocytes	Mice model	Provided a local sustained release of verteporfin and significantly maintained cartilage homeostasis in a mice OA model	[72]
Microspheres	Microencapsulation of human osteoarthritic chondrocytes (hOACs)	Collagen	Collagen scaffold	hOACs	—	Collagen microspheres, as a screening platform, better maintained the hOAC phenotype compared with the 2D monolayer and 3D pellet cultures	[73]
Microspheres	As a scaffold	Cartilage	Cartilage microspheres	Rabbit MSCs	—	Induced the in vitro chondrogenesis without adding any serum or induction components	[74]
Nanocapsules	Celecoxib delivery	HA	—	—	Rat model	Spherical shape, high entrapment efficiency (97.98%), prolonged drug release, and improved histopathology analysis	[65]
NPs	Delivery of SM	PLGA	—	—	Rat model	Increased the chondroprotective effects of SM	[68]
NPs	KGN delivery	PLGA	m-HA	—	Porcine model	Improved hyaline cartilage and subchondral bone repair and demonstrated better therapeutic efficacy in full-thickness chondral defects	[71]
NPs	Melatonin delivery	Albumin	PCL scaffold	Human chondrocytes	—	Prolonged the drug release for 22 days and increased GAG deposition	[75]
Nanotubes	Cartilage repair	Carboxylated SWCNTs	SWCNTs/BSA/collagen composite scaffold	BMSCs	Rabbit model	Had no cytotoxic effect on BMSCs, improved mechanical properties and cell proliferation, and repaired cartilage defects in a rabbit model	[76]
Electrospun nanofibers	Scaffold fabrication	ECM/PCL hybrid	Cartilage-derived ECM/PCL composite	Rabbit chondrocyte	Mice model	Considerably promoted the proliferation of chondrocytes in vitro and facilitated the regeneration of cartilage in vivo	[77]
Electrospun nanofibers	Fabrication of a biocompatible scaffold	PLA/gelatin	CS-modified nanofibers	Rabbit BMSCs	Rabbit model	Had appropriate mechanical properties and suitable biocompatibility, showed better chondrogenic differentiation and promoted cartilage regeneration	[78]
Electrospun nanofibers	Fabrication of scaffold	PLLA	The PLLA/PDA/CS membranes	Rabbit chondrocytes/rabbit BMSCs	Rabbit model	Considerably facilitated the filling of the defect site and the generation of hyaline-like cartilage in vivo	[79]
Electrospun nanofibers	Scaffold fabrication	PCL/PEO	PCL/PEO combined with MSCs-derived TE construct	Rabbit synovial stem cells	Rabbit model	Significantly prevented meniscal extrusion, exerted a chondroprotective effect, and repaired meniscal defects	[80]
Nanocapsules	Delivery of TGF-β1	Gelatin and iron oxide	—	ATDC5 cells	—	Magnetic gelatin nanocapsules improved the differentiation of ATDC 5 cells with the increased expression of Col2a1 and aggrecan	[81]
Nanocrystal– polymer particles	Delivery of p38α/β MAPK inhibitor	PLA	—	Human OA synoviocytes	Mice model	Were non-toxic to cultured human OA synoviocytes, exhibited good retention in the joint and adjacent tissues, and also decreased inflammation and joint degradation	[67]
Nanofibers	Fabrication of collagen-like nanorods	Chitosan and polydiisopropyl fumarate	Fumarate copolymer– chitosan crosslinked nanofibers	Rat BMPCs/rat chondrocytes	—	Supported cell attachment and growth, as well as promoted both osteogenic and chondrogenic differentiation	[82]
NPs	Delivery of curcuminoid	HA/chitosan	—	Rat chondrocytes	Rat model	Provided prolonged release of curcuminoid, inhibited NF-kB signaling and the expression of MMP-1 and MMP-13, and upregulated the expression of type-II collagen in chondrocytes in vitro, as well as reduced the Outerbridge classification and Mankin pathological scores in a knee OA model	[69]
Nanogels	Encapsulation of TGF-β3	Alginate	—	hMSCs	—	Significantly reduced burst release, provided the sustained release of TGF-β3, and also resulted in better chondrogenic differentiation of hMSCs	[83]
Nano- composites	Fabrication of scaffold	PLDLA/HAp	PLDLA/HAp enriched with sodium alginate	—	Rabbit model	Improvement in articular cartilage defect treatment	[84]
Dendrimer	Delivery of KGN	PEGylated PAMAM	—	BMSCs	Rat model	KGN-PEG-PAMAM conjugate could induce higher expression of chondrogenic markers	[85]
NPs	Providing high RGD surface density	Gold	—	hMSCs	—	Had a promotive effect on cartilaginous matrix production and marker gene expression	[86]
Dendrimers	Providing a surface for cell attachment	PAMAM	A PAMAM surface with fifth-generation (G5) dendron structure.	hMSCs	—	Affected the expression of type-II and type-X collagens via effects on cell aggregate behavior	[87]
Magnetic NPs	Labeling of chondrocytes	Iron oxide	Collagen-chitosan/PLGA	Rabbit chondrocytes	—	Magnetic nanoparticles did not affect the cell phenotype and provided a technique for tracking cartilage regeneration and osteochondral defect repair	[88]
Nanofibers	Fabrication of nanofiber-based scaffold	PLGA	PLGA	hMSCs	—	Induced MSC differentiation into bone and cartilage	[89]
Nanofibers	Fabrication of scaffold	PLLA-PCL- collagen	PLLA-PCL- collagen/HA	Rabbit MSCs	—	Promoted orientation, adhesion and proliferation of BMSCs as well as expression of chondrogenic markers	[90]
Magnetic NPs	Physical stimuli	Magnetic NPs isolated from Magneto- spirillm sp.	Micromass culture system used	hMSCs	—	Enhanced the level of sulfated glycosaminoglycan (GAG) and collagen synthesis,and facilitated chondrogenic differentiation	[91]
NPs	KGN delivery	Chitosan	—	hMSCs	Rat model	Provided the sustained release of KGN and induced higher expression of chondrogenic markers	[92]
NPs	Co-delivery of Cbfa-1-targeting siRNA and SOX9 protein	PLGA	—	hMSCs	Mice model	Highly expressed chondrogenesis-related extracellular matrix (ECM) components	[93]
Nanofibers	Fabrication of electrospun embedded nanocomposite.	PLLA	PEG-POSS/PLLA	hMSCs	—	hMSCs were able to attach, proliferate, and differentiate into chondrocytes	[94]
NPs	Delivery of pDC316-BMP4-EGFP Plasmid	PLGA	PLLGA	Rabbit ADSCs	Rabbit model	BMP-4 plasmid could be successfully delivered into ADSCs by PLGA nanoparticles and promoted in vitro and in vivo chondrogenesis	[95]
Electrospun nanofibers	Fabrication of scaffold.	PCL	PCL	Human MenSCs	—	Induced chondrogenic differentiation of menstrual blood derived stem cells	[96]
NPs	TGF-β1 gene delivery.	Calcium phosphate	Collagen/chitosan	Rat MSCs	—	Could successfully induce MSC chondrogenic differentiation	[97]
Nanotubes	Providing titanium dioxide (TiO_2_) based surface.	TiO_2_	TiO_2_ nanotube	Limb mesenchymal cells	—	Could support chondrocytic functions	[98]
Nanofibers	Fabrication of scaffold.	PLLA	PLLA	hMSCs	—	Expressed cartilage-specific gene and formed typical cartilage morphology	[99]
Nanofibers	As scaffold	PCL	PCL	hMSCs/pig chondrocyte	Swine model	Showed the most complete repair, generated hyaline-like cartilage tissue, and had the highest equilibrium compressive stress (1.5 MPa) in the regenerated cartilage	[100]

Heparin is a highly attractive natural polysaccharide for incorporation in scaffolds and NPs. Its highly negative charges enable heparin to form an electrostatic interaction with growth factors. Therefore, heparin can be employed as an essential component of NPs to develop a controlled release platform for growth factor delivery systems. In this regard, the complexation of cationic polymers such as Pluronic F68 [56], polyethyleneimine (PEI) [57], and poly-ʟ-lysine [58] with anionic heparin has been widely used to promote ionic interactions for the preparation of growth factor-loaded NPs. Heparin-functionalized NPs could potentially yield a sustained release of growth factors over several weeks [57], in addition to improved stability of the growth factor structure and function [58], both of which are necessary properties for cartilage TE programs.

Growth factor supplementation for the in situ differentiation of MSCs may be a challenging area, which can be addressed with the use of growth factor-loaded NPs. For instance, the in situ chondrogenic differentiation of human adipose tissue derived stem cells induced by TGF-β1-loaded NPs was similar to that of in vitro predifferentiated and implanted chondrocytes after three weeks [59]. The in vivo chondrogenesis analysis of rabbit bone marrow derived stromal cells (rBMSCs) seeded in fibrin hydrogel containing TGF-β3-loaded NPs showed that this system provided a sustained level of growth factor for a long time (28 days), which allowed for the formation of hyaline cartilage [58].

In addition to providing a scaffold with controlled release properties, the incorporation of NPs eliminates the need for external and intermittent supplements used to develop nanocomposites and enhances the mechanical properties of hydrogels. The fabrication of a photopolymerized maleilated chitosan hydrogel containing micro-/nanoparticles of methacrylated silk fibroin, a natural fibrous protein mimicking the collagen structure, improved the mechanical properties of nanocomposites fabricated for cartilage TE [60]. The development of a magnetic nanocomposite hydrogel from poly(vinyl alcohol) (PVA), nanoscale hydroxyapatite, and magnetic NPs (Fe_2_O_3_) has also shown that the incorporation of NPs enhances the mechanical properties of the scaffold and regulates the behavior of cells seeded in the nanocomposites [61]. A magnetic hydrogel-based nanocomposite can provide a promising scaffold for cartilage tissue regeneration because it can respond to external magnetic fields and specifically relocate to the defective cartilage to resurface the injured site [62].

Initial OA is a slow, complex process characterized by the inflammation and subsequent production of cartilage degradation enzymes by chondrocytes [63–64]. Anti-inflammatory drugs can be used as a primary strategy to suppress inflammatory cytokines to ameliorate OA, particularly inflammatory arthritis. For instance, celecoxib-loaded hyaluronan nanocapsules showed excellent potential in the management of osteoarthritis by prolonging the drug residence time. The intra-articular administration of these nanocapsules in a rat model of OA remarkably decreased knee swelling and improved cartilage repair [65]. In another study, to suppress the expression of pro-inflammatory cytokines in inflamed cartilage, researchers designed poly(NIPAm-AMPS) NPs for the delivery of cell penetrating anti-inflammatory peptides, which selectively target defective sites [66]. In this study, the results showed that an anti-inflammatory peptide-loaded NP could selectively and effectively reduce the expression of pro-inflammatory cytokines within the injured cartilage [66]. On the other hand, p38 MAPK inhibitor-loaded nanostructures exhibited excellent retention at the target site for up to two months and decreased inflammation and joint destruction in two different mouse models [67]. Silymarin (SM)-loaded PLGA NPs were investigated for improving monoiodoacetate-induced osteoarthritis in rats. The SM-loaded NPs showed suitable properties in terms of particle size (81.4 nm), zeta potential (−28.3 mV), and high encapsulation efficiency (97.5%). Histological criteria, knee bend score, and oxidative stress remarkably improved in rats treated with the SM-loaded NPs. The results of this study showed that the encapsulation of SM in PLGA NPs enhanced its chondroprotective effects by improving the stability and bioavailability of the SM [68]. Curcuminoid-loaded HA-chitosan NPs (HA-CNPs) have demonstrated excellent potential in OA treatment due to providing an ideal drug loading capacity (38.44%) and prolonged drug release [69]. The results showed that the curcuminoid-loaded HA-CNPs suppressed the NF-kB pathway and decreased the expression of MMP-1 and MMP-13 while increasing the expression of type-II collagen in a knee OA chondrocyte model. Furthermore, the administration of these NPs remarkably decreased the Outerbridge classification and Mankin pathological scores in rats with knee OA [69]. Recently, researchers developed an injectable hyaluronic acid hydrogel (m-HA) containing kartogenin (KGN)-loaded PLGA NPs and subsequently evaluated the preclinical efficacy of this system on full thickness osteochondral defects in a porcine model [70–71]. The results indicated that this system facilitated the filling of the defects and subchondral bone tissue repair as well as providing better therapeutic efficacy in full thickness chondral defects through the controlled and sustained release of KGN [71].

**3.1.2.2 Nanofibers.** Nanofibers are a versatile class of nanomaterials characterized by two nanoscale dimensions and a third larger dimension (below 1000 nm) [33,101]. Their biomimetic properties and simplicity of fabrication make nanofibers potential candidates for biomedical applications (Table 1). Collagen fibril and fibrous proteins are naturally occurring nanofibers whose fiber diameters range between 50 and 150 nm, depending on tissue type and function. Various techniques to fabricate nanofibers include 3D printing, molecular self-assembly, electrospinning, weaving, phase separation, and template synthesis [102]. Electrospinning is widely used given its simplicity, cost effectiveness, unlimited substrate use, appreciable surface to volume ratio, tunable porosity, and ability to generate nanofibers over a wide range of sizes and shapes [102]. Electrospun nanofibers are widely used in various fields from industry to biomedicine because of their excellent characteristics. Electrospun nanofibers have been used in medicine as wound dressings [103], medical textile compounds [104], drug delivery systems [105], and in regenerative medicine and TE as scaffolds to regenerate different tissues and organs.

The surface of electrospun nanofibers, which are excellent biomaterials to fabricate TE scaffolds capable of forming ECM mimicking structures can be physicochemically modified to meet the biocompatibility requirements of nanofiber biomaterials. For example, to modify the surface of nanofiber polymers, plasma treatment has been used to provide them with hydroxy, carboxyl, or amine polar groups to improve physical properties such as wettability, polarity, and bioadhesion [102]. As a result, surface-modified nanofibers will more likely absorb bioactive molecules such as growth factors. Thus, the sustained release of bioactive molecules from scaffolds will be achieved.

Electrospun nanofibers can be synthesized from either natural or synthetic polymers and used for lineage-specific differentiation of MSCs. Multi-lineage differentiation of MSCs in a nanofiber scaffold fabricated from polycaprolactone (PCL) has shown that electrospun nanofibers can be potentially used to provide a scaffold substrate for adipogenic, osteogenic, and chondrogenic differentiation using specific differentiation media [106]. It was observed that the architecture of a scaffold has a profound effect on the preferential lineage-specific differentiation of MSCs. Mellor et al. investigated the multi-lineage differentiation potential of electrospun nanofibers in comparison with the 3D printed scaffold of PCL. They indicated that PCL nanofibers might provide a better architecture for chondrogenic differentiation while PCL-based 3D printed scaffolds might be more appropriate for osteogenic differentiation [107].

**Figure 3:**
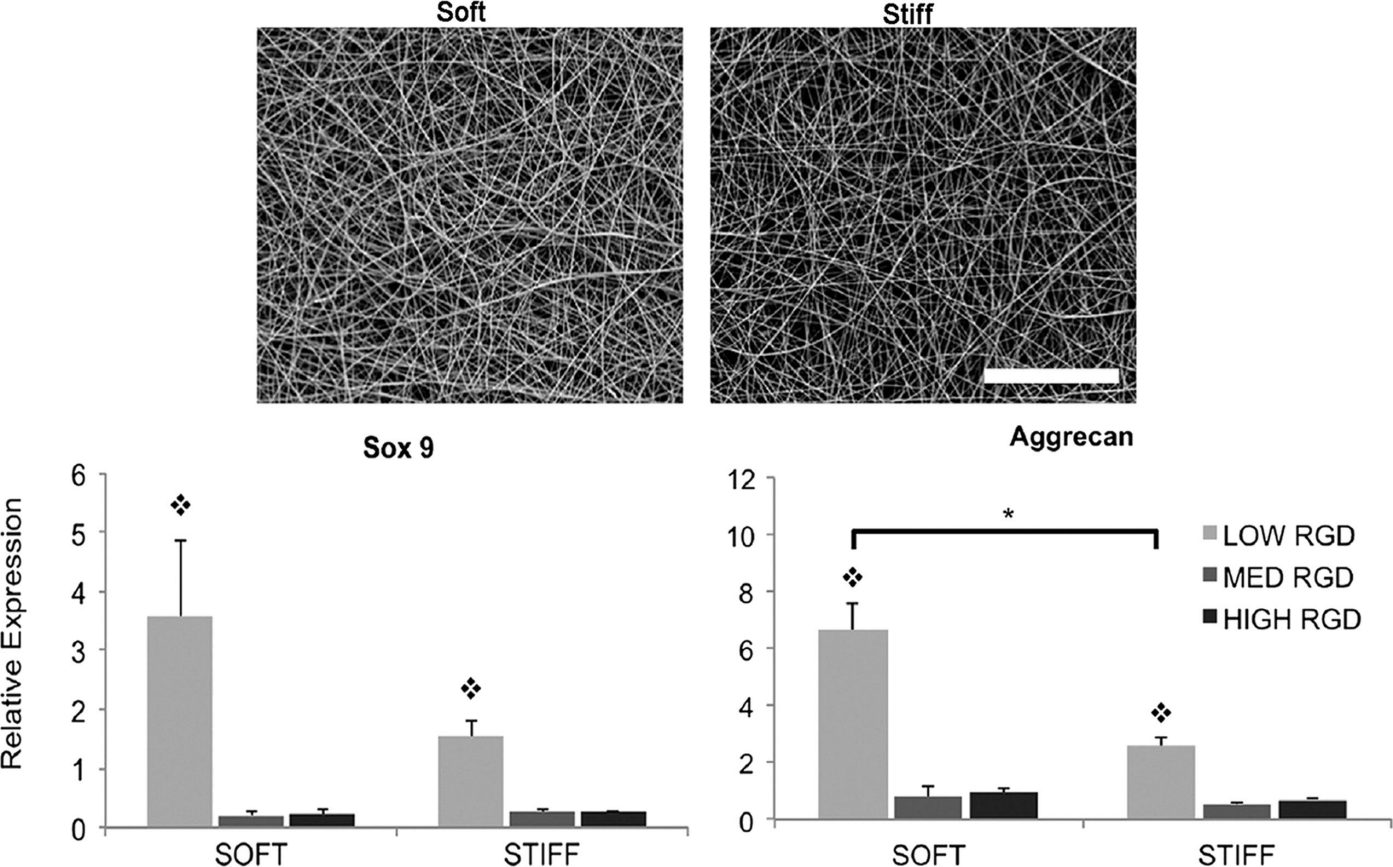
(Top) SEM images of electrospun nanofibers, representing the soft group (35% modified MeHA) and the stiff group (100% modified MeHA). (Bottom) Gene expression analysis of chondrogenic markers of hMSCs seeded on these scaffolds. RGD peptides in different concentrations were also used to enhance scaffold adhesivity. Figure 3 was reprinted from [8], Biomaterials, vol. 34, by I. L. Kim; S. Khetan; B. M. Baker; C. S. Chen; J. A. Burdick, “Fibrous hyaluronic acid hydrogels that direct MSC chondrogenesis through mechanical and adhesive cues”, pages 5571–5580, Copyright (2013), with permission from Elsevier. This content is not subject to CC BY 4.0.

The actual behavior of chondrocytes in a native microenvironment is a consequence of multiple factors, the interactions of which regulate the cell responses. Among these, the overall 3D environment may have a greater influence on chondrocyte activity and the synthesis of cartilage ECM. The choice between microscales and nanoscales required for the development of scaffolds highly depends on the nature of the scaffold substrate as well as the adopted architecture and tailoring method for the construction of the scaffold. Researchers have assessed the effects of different morphologies of a polylactide (PLA) fiber-based scaffold [microfiber scaffold (MS) and nanofiber-coated microfiber scaffold (NMS)] on the behavior of human articular chondrocytes [108]. Although the results showed no appreciable difference between NMS and MS scaffolds in terms of inducing redifferentiation, nanoscale patterning of the microfibers influenced cell proliferation. In another study, similar results were obtained regarding the effect of poly(ʟ,ᴅ-lactide) (PLDLA) microfibers or films (nanofiber composites) coated with PLDLA nanofibers on the behavior of bovine chondrocytes [109]. The researchers observed that electrospun nanofibers supported the adhesion of cells and maintained the chondrocyte phenotype. Besides, the adhesivity and mechanics of the nanofiber-based scaffolds could be tuned to meet the needs of chondrocyte behavior and function. Kim et al. studied the effects of adhesivity and mechanics of electrospun nanofibers of HA on hMSC chondrogenesis [8]. They synthesized photocrosslinkable RGD (Arg-Gly-Asp)-engrafted HA for electrospinning. The RGD peptide density has been used to tune nanofiber adhesivity, and the intrafiber crosslink density has also been used as a parameter to calibrate nanofiber mechanics [8]. Cell spreading, proliferation, and cytoskeletal organization depended on the RGD density. Meanwhile, the expression of chondrogenic markers was concomitantly regulated by fiber mechanics and adhesivity such that softer fibers and lower RGD densities improved chondrogenesis (Figure 3).

In addition to providing the building blocks for the construction of fibrous scaffolds, nanofibers can be used as promising reinforcing agents to enhance the mechanical properties of hydrogels. Nanofiber-filled nanocomposites can also have different functions, depending on the nature and concentration of incorporated nanofibers. Incorporating electrospun silk fibers into chitosan/glycerophosphate hydrogels can reinforce the mechanical properties of the chitosan hydrogel and improve the expression of the chondrogenic phenotype [110]. Fragmented PLA fibers incorporated into HA-grafted alginate hydrogel have been shown to increase the compressive modulus by approximately 81% compared to the nanofiber-free hydrogel scaffolds. Moreover, they maintained the round phenotype of the encapsulated chondrocytes and improved the production of cartilage-specific markers [111]. Furthermore, two or more nanofiber-based components can be blended and used for electrospinning to obtain composite mats or fabricate nanofiber-incorporated composites. In this regard, a poly(ʟ-lactic acid) (PLLA)/silk fibroin (SF) nanofibrous scaffold fabricated via electrospinning was investigated for its chondrogenic potential [112]. The results showed that the composite scaffold could facilitate chondrocyte adherence, proliferation, and secretion of cartilage-specific ECM as well as maintaining the phenotype stability compared to flat tissue culture polystyrene surface (TCPS) and PLLA scaffold. It is well-accepted that polysaccharides containing sulfate groups can mimic the physicochemical and biological properties of sulfated GAGs [113]. In this context, Irani et al. used sulfated alginate as a heparin-mimicking polymer and fabricated a 3D electrospun mat through electrospinning of PVA and sulfated alginate solutions. The results demonstrated that human bone marrow (hBM) MSCs attach appropriately and spread on the resulting nanofibrous mats. In addition, the results of type-II collagen expression and immunocytochemistry analyses revealed the chondrogenic differentiation of hBM‐MSCs on alginate sulfate nanofibrous mats [114].

There are promising reports on the in vivo application of nanofiber scaffolds to repair osteochondral defects in animal models (Table 1). For example, in a comparative study, a composite mat of PVA and chondroitin sulfate (CS) nanofibers was prepared using a wet electrospinning system for the in vitro chondrogenesis and in vivo analysis of the effect of acellular fiber scaffolds on articular tissue repair in a rat osteochondral defect model [115]. The CS/PVA nanofiber scaffold increased the expressions of chondrogenic markers and appreciably repopulated the defective cartilage site compared to pellet culture and PVA fibers. In line with this, the application of a resveratrol–PLA–gelatin scaffold in a rat articular cartilage defect model promoted the repair of cartilage injury and had a faster repair rate compared to a PLA–gelatin nanoscale scaffold [116]. Furthermore, to enhance biocompatibility, a 3D porous nanofiber scaffold made of gelatin and PLA was modified using CS [78]. The results demonstrated that the modified scaffold had appropriate mechanical properties and biocompatibility. BMSCs seeded on this scaffold showed better chondrogenic differentiation compared to cells seeded on the PLA–gelatin scaffold. In a rabbit cartilage defect model, the CS modified 3D nanofiber scaffold showed anti-inflammatory effect and promoted cartilage regeneration [78]. To accelerate the regeneration of cartilage tissue, researchers successfully designed and fabricated aligned porous PLLA electrospun fibers with biomimetic surfaces by combining the electrospinning process with the polydopamine (PDA) coating approach. The in vivo results of this study in a rabbit cartilage defect model showed that the PLLA/PDA/CS fibers considerably facilitated the filling of the defect site and the regeneration of hyaline-like cartilage [79]. The implantation of the hBMSCs-laden PCL nanofibrous scaffold in a swine model with 7 mm full thickness cartilage defects showed the most complete repair and generated hyaline-like cartilage tissue in comparison with the acellular construct and the no-implant group [100]. In another study, researchers fabricated a cartilage-derived ECM/PCL hybrid nanofibrous scaffold to improve the biological functionality of PCL nanofibers. The presence of cartilage-derived ECM nanofibers in the hybrid nanofibrous scaffold considerably promoted the proliferation of chondrocytes in vitro and facilitated the regeneration of cartilage in vivo [77].

Articular cartilage function greatly depends on the structural architecture and integrity of the essential components of the ECM, which resemble a pseudostratified structure. It is believed that the presence of a gradient concentration distribution of bioactive molecules through articular cartilage tissue has a profound effect on the formation of a multilayered structure within it. In the wake of the construction of a bioinspired scaffold, interesting and innovative nanofiber-based scaffolds have been developed using electrospinning methods in which the concentration distributions of two bioactive agents were applied [117] or a range of membranes with different proportions of components were prepared to mimic the zonal matrix of the bone–cartilage interface [118].

**3.1.2.3 Nanotubes.** Nanotubes are a particular class of nanostructured compounds with a diameter of 1–100 nm known for their cylindrical geometry. Nanotubes are used in a wide variety of nanotechnology-related areas, with recent widespread applications in biomedicine and biotechnology research. Nanotubes can be classified into two major categories depending on their structure, namely single-wall or multiwall nanotubes [119]. They consist of either a single sheet of atoms or a multilayer wrapped to form a hollow core. They are preferably made from different compounds such as carbon, silicon, boron, and halloysite clay sheets and possess unique physicochemical properties.

Among nanotube structures, much attention has been paid to carbon nanotubes (CNTs) because of their excellent mechanical and tensile strength properties, thermal and electrical conductivity, and high surface ratio. CNTs were discovered in 1991 by Sumio Iijima [120] and have been widely used in different biomedical areas including cellular imaging [121], biosensor development [122], bioactive molecular delivery [123], and, in particular, TE. To provide scaffolds with improved mechanical strength and innovative properties, a small fraction of CNTs can be dispersed as fillers in composites. However, application and integration of CNTs with polymers have encountered technical limitations because of the low dispersity of CNTs within a wide range of solvents. CNTs are chemically highly inert molecules and need to be functionalized through either harsh acid treatment or bioconjugation to increase their biocompatibility. It has been reported that COOH-functionalized single-wall carbon nanotubes (SWCNTs) dispersed in hMSC media have the least toxicity to cells without adverse effects on the adipogenic, osteogenic, and chondrogenic potentials of the hMSCs [124]. However, the migration of SWCNTs through the cell wall to the nucleus was detected using fluorescence-labeled CNTs.

In line with these results, the long term exposure of chondrocytes to COOH- and PEG-functionalized SWCNTs in 2D cultures, 3D pellet cultures, and nanocomposite scaffolds showed no detrimental effects on the chondrocyte viability and promoted the expression of articular cartilage-specific ECM proteins [125]. In addition, functionalized SWCNTs improved the biomechanical properties of cell-laden nanocomposite structures compared with the control group [125].

One of the pioneering studies on the pathological complications arising from implanting CNT incorporated alginate hydrogel was carried out by Kawaguchi and co-workers. They reported a mild inflammatory response and non-cytotoxic effects following the implantation of the scaffold in a rat model [126]. Zadehnajar et al. incorporated 0.5 wt % multiwalled carbon nanotubes (MWCNTs) into an electrospun PCL–gelatin (70/30) scaffold and evaluated its influence on the physical, chemical, and mechanical properties as well as cell response [127]. The addition of 0.5 wt % MWCNTs into the scaffold led to a decrease in the average fiber diameter, an increase in hydrophilicity and tensile strength, without adversely affecting its porosity percentage and mechanical strength. Moreover, the results showed that the presence of COOH-functionalized MWCNTs as a reinforcement in the scaffold had no adverse effect on the behavior of chondrocytes [127]. In another study, researchers developed a kind of CNT-based composite scaffold to investigate the effect of COOH-functionalized SWCNTs on the repair of cartilage defects in a rabbit model [76]. The results showed that the addition of the COOH-functionalized SWCNTs not only improved the mechanical properties and cell proliferation, but also had no cytotoxic effect on BMSCs. The experimental results of implanting the composite scaffold demonstrated that reasonable addition of SWCNTs is critical to repair the cartilage defects and the 0.5% group had the best repair effect compared with 1.0% and 2.0% groups [76].

Numerous studies evaluated the application of CNTs for structural reinforcement of scaffolds and biomaterials, which have shown promising properties for TE of neural, cardiac, vascular, and bone tissue. Application of CNTs can be beneficial because they are not biodegradable and provide a more stable matrix in which osteoblasts can proliferate and deposit new ECM. The integration of CNTs into a polymer matrix would increase surface roughness and activate the mitogen-activated protein kinase (MAPK) pathway [128], which is responsible for regulating the osteogenic response of cells to surface roughness. In this regard, the fabrication of a CNT-incorporated PLGA nanocomposite has resulted in the enhanced surface roughness and increased attachment and proliferation of MC3T3-E1 osteoblasts [129]. In vitro osteogenesis analysis also showed a significantly higher rate of differentiation by incorporation of CNTs into the PLGA scaffold.

**Figure 4:**
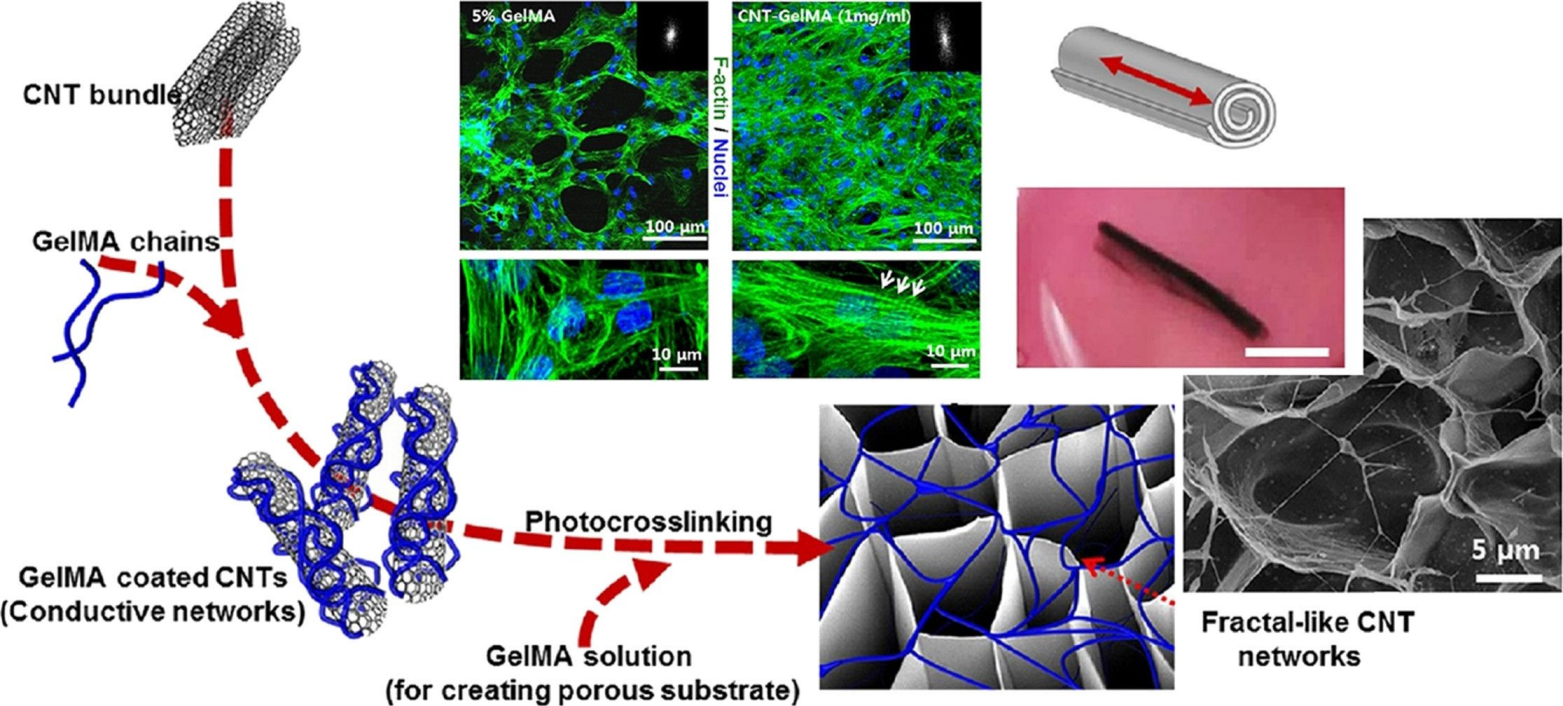
Schematic representation of GelMA-coated CNTs embedded in gelatin hydrogel for the preparation of the engineered cardiac tissue. Confocal images revealed that cardiomyocytes cultured on 1 mg/mL CNT-GelMA hydrogel had a more uniform cell distribution relative to cells cultured on control GelMA hydrogel. Schematic representation of a tubular actuator and its beating direction (red arrow) in addition to an optical image of a sample. Figure 4 was reprinted with permission from [132], Copyright (2013) American Chemical Society. This content is not subject to CC BY 4.0.

Most studies regarding CNT-incorporated nanocomposites have reported enhanced mechanical strength and biocompatibility. Although this mostly arises from the considerable structural rigidity of CNTs, the interaction of CNTs with the material context should not be ignored. Functionalized CNTs can physically interact with some of the chemical groups on polymers and facilitate the dispersion of CNTs in composites. For example, it has been reported that COOH-functionalized SWCNTs are readily embedded into type-I collagen scaffolds and developed scaffolds with enhanced mechanical and functional properties [130]. In line with this, CNTs coated with methacrylated gelatin (GelMA) reinforced the mechanical properties of hybrid microgels without inhibiting cell growth [131]. The researchers also engineered cardiac patches using a cardiomyocyte-incorporated CNT–GelMA hydrogel for TE of the cardiovascular system [132]. To fabricate CNT–GelMA nanocomposites, they added MWCNTs into GelMA to improve the electrophysiological and mechanical properties. The electrical conductivity of CNTs allowed the researchers to form nanocomposite 3D actuators with tunable linear contractile actuation and cardiac pumping ability (Figure 4). This unique property of CNTs was also used to improve the functions of chondrocytes encapsulated in a polycarbonate urethane nanocomposite in which electrical stimulation led to enhanced cell densities [133].

CNTs are usually used as fillers in combination with other compounds in the construction of nanocomposites. However, there are a few reports about the fabrication of nanofilms of vertically aligned (VA) CNTs for the 2D culture of chondrocytes. To simultaneously introduce polar functional groups (COH, COOH, and OH) and surface roughness to CNT, Antonioli et al. used oxygen plasma treatment and fabricated novel superhydrophilic VA–CTN films. This treatment could increase the wettability of the nanofilms to acquire appreciable cytocompatibility [134]. The chondrocytes expressed major chondrogenic markers upon cultivation on these scaffolds. To treat osteochondral lesions, Stocco et al. developed porous PDLLA/VA-CNTs/nanohydroxyapatite (PDLLA/CNT/nHA) scaffolds using recent methodologies [135]. The results showed that this nanocomposite supported the viability of chondrocytes and decreased the expression of type-I collagen mRNA. The study reported that the addition of CNT/nHA formed a porous scaffold, which was advantageous for cell growth [135]. In another study, researchers fabricated nanocomposite scaffolds through the combination of CNTs with PCL nanofibers to reinforce them for knee meniscus tissue engineering. The results demonstrated that the incorporation of CNTs into PCL scaffolds improved the mechanical properties, such as failure stress, yield stress, and Young’s modulus, and had no adverse effects on MSC survival [136]. Due to the specific orientation of chondrocytes, tissue engineering faces a limitation to mimic the architecture of cartilage. Therefore, Janssen et al. used VA–MWCNT micropillars to induce an unidirectional orientation of chondrocytes [137]. The Young’s modulus of the VA–MWCNT micropillars was in the range of the natural ECM of articular cartilage. The proliferation of chondrocytes to achieve unidirectional orientation was controllable by optimizing the size and spacing of VA–MWCNT micropillars. Cell attachment, proliferation, and ECM production reportedly were enhanced by micropillars in comparison to the control groups [137].

Apart from carbon nanotubes for TE, there are other nanoscale tube-based compounds, which can potentially be used for biomedical purposes. Among these, silica nanotubes have the greatest potential for integration in biomaterials because of their biocompatibility, photoluminescence activity, and ease of surface modification. Silica nanotubes exhibit considerable results regarding bone TE due to their ability to provide a surface with proper roughness, which facilitates osteoblast attachment and proliferation, and can provide an active site for the deposition of inorganic calcium apatite [138].

In addition to silica, titanium dioxide (TiO_2_) nanotubes have recently attracted widespread attention in the biomedical field due to the great biocompatibility, large specific surface area, and the provision of an inner space capable of being filled with drugs and bioactive molecules [139–140]. Recently, TiO_2_ nanotubes have been introduced as potential candidates for bone TE due to osteoblast attachment and corrosion resistance [141]. Wan et al. used bacterial cellulose as a template to synthesize calcined mesoporous TiO_2_ nanotubes and constructed a nanotube-based scaffold using a template-assisted sol–gel method [142]. In addition to enhancing the ALP activity and mineralization, the scaffold showed improved cell proliferation. Khoshroo et al. fabricated a 3D PCL/TiO_2_ sintered microsphere scaffold, which improved the mechanical and biological properties for bone TE purposes [143]. Moreover, the results showed that the presence of TiO_2_ nanotubes enhanced the biocompatibility and bioactivity of the PCL microspheres and provided an excellent biomaterial for cell differentiation and mineralization.

In recent years, numerous efforts have been made to evaluate new candidate biomaterials to bridge the gap between material sciences and biomedicine, in particular, TE. However, nanotube studies have been mainly limited to CNTs. Nanotube arrays such as boron nitride nanotubes (BNNTs) and halloysite nanotubes (HNT) have not been investigated despite their superior physicochemical attributes and structural properties similar to CNTs. For example, poly(propylene fumarate) (PPF) nanocomposites containing BNNTs showed increased mechanical reinforcement, higher adsorption of collagen I protein, excellent cell attachment as well as ECM deposition compared to the PPF control [144]. Halloysite clay, a natural aluminosilicate material, is an inexpensive and abundant inorganic compound, which can be obtained from mines. Aluminosilicate layers can be rolled into a hollow cylinder structure to form tube structures less than 100 nm in diameter. Halloysite is a biocompatible nanomaterial, which does not need to be functionalized and can be a potent alternative for CNTs in biomedical applications [145]. Bonifacio et al. have developed a hydrogel nanocomposite scaffold composed of gellan gum and glycerol and reinforced by halloysite nanotubes for skin TE [146]. Integration of 25% HNTs into gellan gum reinforced the mechanical properties of the hydrogel and increased biocompatibility when human dermal fibroblasts were seeded on the scaffold or encapsulated in the hydrogel. Incorporation of HNTs into alginate hydrogel also yielded enhanced mechanical properties and scaffold density [147]. HNTs led to a decreased water swelling ratio of alginate, increased attachment of the fibroblast cells, and improved stability of alginate scaffolds against enzymatic degradation. In line with this, a nanocomposite scaffold composed of blended polymers of chitosan–gelatin–agarose and incorporated by 3–6 wt % HNTs was fabricated by Naumenko et al. using freeze-drying. This scaffold was evaluated by in vitro as well as in vivo studies [148]. The authors reported enhanced mechanical properties of the scaffold. The in vivo study showed the biocompatibility of the scaffold in the implanted animal with a slight inflammatory effect without implant rejection.

#### 3.2 Biological cues in cartilage TE

Natural ECM provides a desirable microenvironment containing biologically important motifs (Table 2) for cell attachment, spread, migration, proliferation, and differentiation [149]. This microenvironment is inevitably disregarded during the in vitro cell culture and expansion.

**Table 2:** Summary of the factors influencing cell fate that should be taken into account when designing a scaffold.

Factors	Example	Effects/applications	Important notes	Ref.

Biological cues	Full-length proteins, cell adhesion motifs, HA binding domain, ECM constituents, etc.	Biological cues provide a desirable microenvironment for cell attachment, spread, migration, proliferation, and differentiation. The local density of RGD supports cell adhesion and condensation and regulates early chondrogenic differentiation of MSCs. Cell adhesion motifs are used to enhance cell motility and hydrogel adhesion. ECM components of MSCs can induce MSC proliferation, migration, and multilineage differentiation. MSC-derived ECM can provide a proper substrate for expansion, proliferation, and maintenance of the chondrogenic phenotype.	The composition of the MSC-derived ECM greatly depends on the stage of the chondrogenic differentiation of MSCs. MSC-derived ECM has an undefined composition and may elicit unwanted immunological responses or cause an improper cell commitment.	[149–152]
Surface chemistry	—	Surface chemistry plays an important role in cell adhesion, proliferation, and morphology. Surface chemistry can control phenotype and promote the expression of lineage-specific markers in differentiated MSCs.	—	[153–157]
Geometry	Curvature, porosity, pore size, and pore shape.	Scaffold geometry plays a key role in chondrocyte adhesion and regulates their phenotype and function. The geometry regulates the orientation of the tubulin and actin cytoskeleton and affects the mechanical behavior and Ca^2+^ signaling of chondrocytes. The tissue regeneration rate is proportional to the surface curvature and could be significantly influenced by the pore shape. The chondrocyte density considerably increases with scaffold pore size and porosity, while ECM synthesis significantly decreases. The pore diameter correlates negatively with the metabolic and anabolic activities of chondrocytes. An effective strategy to promote chondrogenesis at the beginning of in vitro cartilage engineering is the combination of small pores with low porosity.	While other parameters of the porous structure are fixed, the change in pore size often leads to a change in the mechanical properties of the porous scaffold.	[158–162]
Microtopography	Microgrooves, microgrids, microholes, and micropillars.	Cell structure, morphology, and migration are affected by microtopography. The depth, width, and direction of microtopography influence migration rate, directional movement, and size of aggregates. Microtopography can promote MSC growth and differentiation. A microgroove topography can retain the phenotype of chondrocytes and improve their adhesion and proliferation.	The size of the ridges plays a more important role than the grooves in determining the MSC fate. The impact of surface topography on cell behavior strongly depends on surface chemistry.	[163–168]
Nano-topography	Nanogrooves, nanogrids, nanoholes, and nanopillars.	Nanopatterned surfaces can induce and enhance receptor-mediated cellular responses. The nanoscale topography could promote epigenetic changes.	The nanoscale topography alone cannot significantly improve the chondrogenic differentiation and its impact depends on surface chemistry.	[169–172]
Surface stiffness		Surface stiffness arises from substrate chemistry and controls stem cell differentiation. Mild stiffness would induce the ROCK pathway that is responsible for the promotion of the chondrocyte phenotype. The combination of surface stiffness and exogenous TGF-β can support the redifferentiation capacity of chondrocytes to generate cartilage.	There is no widely accepted value for stiffness modulus that specifically determines the fate of stem cells.	[173–176]

Cell culture in 3D matrices, unlike 2D cultures, provides the opportunity to include and recruit the necessary bioactive molecules for proper cell function [177]. In addition to biopolymers, which provide structural integrity and stability, ECM consists of protein motifs and full-length proteins traditionally incorporated in scaffolds to provide proper cell–matrix interactions for tissue regeneration [149]. Among these, cell adhesion motifs including RGD peptide and fibronectin fragments at the nanoscale are widely used to enhance the cell motility and adhesion functionality of hydrogels [178]. For example, a study of the effect of RGD on cell adhesion using nanopatterned RGD-conjugated polyamidoamine dendrimers has shown that the cell adhesion efficiency is directly related to the density of nanopatterned RGD [150]. Interestingly, the evaluation of the impact of RGD dendrimers on the chondrogenic differentiation potential of adult hMSCs demonstrated that the local density of RGD supported cell adhesion and condensation and regulated early chondrogenic differentiation of MSCs [151].

In addition to protein motifs, the incorporation of ECM constituents into the scaffold structure provides numerous advantages over conventional TE approaches. For HA, this could be obtained via the conjugation of the HA binding domain to a polyethylene glycol (PEG) hydrogel, which would provide HA affinity for the PEG hydrogel and consequently improve cartilage tissue production [179]. Given the generally conserved nature of ECM components among species as well as the ability to provide biologically important cues, it is a great benefit to use a decellularized ECM matrix to fabricate ECM mimetic scaffolds. When human chondrocytes were cultured on acellular ECM scaffolds derived from porcine menisci, their cell proliferation and synthesis of the ECM were enhanced. In vivo analysis showed a minimal inflammatory response in hMSC chondrogenesis [180]. Yin et al. used cartilage ECM derived from goat to prepare a cell carrier for chondrogenic differentiation of hMSCs and cartilage formation [181]. They reported that microcarriers could be developed into microtissues enabling MSC differentiation, which could subsequently be used for in vivo cartilage repair.

In addition, ECM derived from MSCs was used to coat polystyrene plastic tissue culture surfaces and led to dramatically increased MSC proliferation, migration, and multilineage differentiation [149]. In line with these results, MSC-derived ECM provided a considerable substrate for expansion, proliferation, and maintenance of the chondrogenic phenotype in the 2D culture system. In vitro as well as in vivo analyses have shown robust cartilage formation by chondrocytes encapsulated in a scaffold constructed of MSC-derived ECM [182]. However, the composition of the MSC-derived ECM greatly depends on the stage of the chondrogenic differentiation of MSCs. This should be taken into account when an ECM based scaffold is used [152].

ECM-derived scaffolds have an undefined composition containing numerous protein domains and motifs as well as different antigens, which may elicit unwanted immunological responses or cause an improper cell commitment [183–184]. For this reason, the fabrication of ECM mimetic scaffolds is more popular for osteochondral TE applications. Substantially more self-assembled scaffolds have been constructed with partially modified biological cues by bottom-up approaches. Incorporation of proteins such as growth factors in the scaffold structure is preferable to traditional administration routes due to their short biological half-lives and rapid degradation in biological fluids [185].

Physical entrapment and chemical immobilization are two distinct strategies used for the presentation of growth factors in TE approaches. From a simple point of view, physical entrapment can imply simple encapsulation of a growth factor during hydrogel fabrication – a process, which lacks efficient and sustained protein release. However, this approach can be pursued by the establishment of physical interactions between the scaffold structure and growth factors. For example, BMP4 could be successfully immobilized on a hybrid hydrogel of collagen/PLGA by protein engineering to conjugate a collagen binding domain (CBD) to BMP4. CBD–BMP4 has a high affinity for the collagen/PLGA scaffold when encapsulated in the hydrogel and shows enhanced osteoinductive activity [186].

In recent years, affinity-based growth factor delivery systems have been successfully used for the physical entrapment of growth factors in scaffolds. Most studies used heparin because of its advantageous biological properties. Approximately one quarter of TGF-β superfamily bound to heparin [187] and heparin-based hydrogels potentially provides an excellent surface for adhesion and proliferation of hMSCs [188]. Heparin can be chemically modified to be covalently co-polymerized with biomaterials without affecting its affinity properties. Heparin has been efficiently integrated into hydrogel structures such as alginate, PEG, hyaluronate, and dextran for affinity-based growth factor delivery of FGF2, BMP2, and vascular endothelial growth factor (VEGF) in osteogenic and chondrogenic differentiation of MSCs [189–196].

#### 3.3 Topographical cues in cartilage TE

Articular cartilage is an ECM-rich tissue consisting of specialized cells surrounded by a mixture of various macromolecules, which provide a 3D structure for physical contact between the resident cells and peripheral material. Apart from the chemical composition of ECM, its morphology and geometrical properties considerably affect the commitment and fate of the resident cells [163]. The so-called topographies refer to the physical cues on the nanometer or micrometer scale. These physical cues have a deterministic effect on cell behavior according to the topography size [197]. Cells can sense topographic patterns ranging from 10 nm to 100 µm [198–199]. The topographies on the nanometric scales usually evoke cellular responses, which are restricted to the re-organization of surface receptors, particularly integrin [169]. On the micrometer scale, the fundamental features of the cells (cell structure, morphology, and migration) are affected [164,200]. Numerous researchers have attempted to better understand the effect of topography on cell behavior [201–203]. Harnessing these results can help researchers tailor new biomaterials for TE purposes (Table 2).

**3.3.1 Microtopographical cues in cartilage TE.** Chondrogenesis is a complex phenomenon, which begins with the establishment of cell–cell contact and cell–ECM adhesion via mechanisms of mechanotransduction [204–205]. Cell collision and condensation are prerequisites for chondrogenesis. In vitro stimulation or facilitation of cell aggregation can assist the development of chondrogenesis. In this regard, the in vitro application of an artificial micrometric topographical cue can improve our understanding of chondrocyte aggregation and cell–ECM interactions. A study that aimed at assessing the effect of microgrooves on the behavior of chondrocyte aggregates [165] showed that microgrooves could induce chondrocyte aggregation. The depth, width, and direction of microgrooves affect the migration rate, directional movement, and size of aggregates.

There is a pervasive problem with the in vitro expansion and proliferation of chondrocytes due to the dedifferentiation of the primary chondrocyte culture. Dedifferentiated chondrocytes are mostly fibroblastic in morphology and fail to express chondrogenic markers such as collagen type II and aggrecan [206–207]. High-density cell seeding in monolayer chondrocyte culture systems has been used to prevent chondrocyte dedifferentiation [208–209]. Of course, it should be noted that in this method, cell proliferation is limited. Previous studies demonstrated that the 3D culture on a microgroove surface topography enabled chondrocytes to retain their phenotypes and improved their adhesion and proliferation [164,166].

Both depth and width of the microgrooves directly influence chondrocyte adhesion and migration. An investigation of primary chondrocyte responses to surface microgroove patterning of sulfated HA on a polyethylene terephthalate (PET) film ranging from 80 nm to 9 mm in depth and 2 to 20 µm in width has shown the enhanced movement of chondrocytes at the 750 nm depth compared to the flat surface. However, the researchers observed that the microgroove structures did not appreciably affect cell spreading/morphology or F-actin organization [166].

In a similar study, two different micropatterned surfaces (grooves with 5 or 25 µm width and 35 nm depth) of hyaluronan on a PET substrate were generated and their effects on chondrocyte cells were evaluated. The results showed that the microgrooves effectively led the growth and alignment of chondrocytes and preserved their phenotype. These results were particularly observed in the 25 μm grooves [164]. In contrast, chondrocytes grown on the plain substrate did not maintain their round phenotype and showed a fibroblast-like morphology. The results of these two studies have indicated that microgroove patterning with sub-micrometer depth could accelerate chondrocyte movement and aggregation. Therefore, although surface chemistry plays an important role in determining cell behavior, topographical cues should also be taken into account in cartilage TE programs (Table 2).

Surface topography can promote MSC growth and differentiation in addition to its profound impact on cell adhesion, migration, and morphology. There are several reports about the recruitment of topographical cues for the induction of multilineage differentiation [167–168,210] or maintenance of MSCs in the undifferentiated state [211].

In an attempt to uncover the potential effect of variation of groove/ridge size on the fate of MSCs, Abagnale et al. designed a microchip to systematically study the in vitro differentiation of MSCs [163]. The micropattern was generated in a polyimide substrate containing an array of 25 different microstructures ranging from 2 to 15 µm in both grooves and ridges. The researchers observed that MSCs were aligned relative to the axis of the grooves and proliferated equally in different microstructures. However, a comparison between the groups indicated that the size of the ridges played a more important role than the grooves in the determination of the MSC fate such that 2 and 15 µm ridges stimulated osteogenic and adipogenic differentiation, respectively. This study emphasized the importance of both microtopography in lineage-specific differentiation of MSCs and the details of the topography when designing a scaffold [163].

Chondrogenic differentiation is a phased process with an expression of early and late chondrogenic markers. ALP and type-X collagen are the most well-known markers of hypertrophy, a natural phenomenon during the terminal differentiation of mature chondrocytes before bone formation [212–214]. Le et al. have selected seven patterns from a library of previously created microtopography platforms, which induced high ALP expression and used them to analyze the chondrogenic differentiation markers of ATDC5 cells [215]. The results showed that most micropatterns inhibited the expression of the early markers while inducing the expression of late chondrogenic markers. These findings demonstrated that, in addition to chemical induction of chondrogenesis, surface architecture could be used to affect the cell behavior and expression of stage-specific markers of chondrogenesis.

**3.3.2 Nanotopographical cues in cartilage TE.** ECM provides a structural framework of microscale and nanoscale structures facilitating cell adhesion, migration, proliferation, and/or differentiation. However, the underlying cellular mechanisms, which sense ECM topography and drive cell behavior, are organized in the nanoscale pattern. Cell receptors, particularly integrins as the most well-defined cell adhesion receptors, which are responsible for sensing and transmitting information about the stiffness of the microenvironment [216], surface chemistry [153], and topography [170], are a few nanometers in size (Table 2). Therefore, it seems that the design and development of bioinspired and biomimetic materials containing nanotopographical cues can provide cell adhesion receptors with robust ligands. Nanopatterned surfaces can induce and enhance receptor-mediated cell adhesion [217], proliferation [171], migration [218], gene expression regulation [219], epigenetic changes [220], and regulation of microRNA expression [221]. The effect of nanotopographical cues on the lineage-specific differentiation of stem cells has been recently observed in various stem cell differentiation programs such as neuron differentiation of human pluripotent stem cells [222], pancreatic differentiation of human embryonic stem cells [223], kidney-derived stem cell differentiation into podocytes [224], and osteogenic differentiation of human dental pulp derived stem cells [172].

A comparison of the cell behavior on nanoscale and microscale surface topographies can lead to considerably different results depending on the cell type and surface substrates. For example, adhesion and differentiation of MSCs on titanium with micrometer- and nanometer-scale grid patterns showed that the microscale grid topography was suitable for cell anchoring and colonization whereas the nanoscale topography was appropriate for cell motility. A superposition of microscale and nanoscale grid topographies strongly intensified cell adhesion. Gene expression analysis showed that the nanoscale topography induced the expressions of osteogenic markers in addition to other differentiation specific markers [225]. However, Skoog et al. observed a higher cell adhesion on a tantalum-coated implant with micrometer and sub-micrometer surface topographies than on a microscale/nanoscale hybrid structure [226]. Yoo et al. demonstrated that a nanogrooved substrate had a profound impact on the re-programming of mouse embryonic fibroblasts (MEFs) to functional induced dopaminergic neurons [227]. Their results indicated that cell alignment was differently affected by the substrate topography. The nanogrooved topography had more impact on cell alignment and geometry both before and after re-programming MEFs. The nanoscale topography significantly induced the expression of dopaminergic markers from re-programmed MEFs compared to the microscale topography, which suggested a probable advantage of nanopatterning for re-programming purposes.

Not only is the scale of surface topography an influential factor on cell anchoring and adhesion, but pattern, feature, and geometry of topography also have critical effects on lineage-specific differentiation of stem cells. It has been found that biomimetic nanotopographical patterns generate a variety of responses from different cells or even a specific cell. Additional study and analysis should be performed to harness the enormous potential of nanotopographical cues for TE purposes [228–230]. In a comparative study, the impacts of three different nanotopographies (nanopillars, nanoholes, and nanogrills) on the chondrogenic differentiation of MSCs were investigated [230]. The results showed that cell structure and morphology as well as proliferation rate of MSCs were differently affected by different topographical patterns. Expression profile analysis demonstrated that nanoholes and nanopillars, unlike nanogrills, promoted chondrogenic differentiation markers.

Topographical cues consist of elements with rough surfaces, which induce a mechanotransduction pathway through the formation of focal adhesion complexes on the cell surface [231]. Integrin receptors play a fundamental role in the localization of cytoplasmic focal adhesion protein-tyrosine kinase (FAK) at the cell–ECM interfaces [232]. Chondrocytes express several members of the integrin receptors, which mediate various functions such as differentiation, matrix re-modeling, response to mechanical stimulation, and cell survival [233]. Thus, a scaffold with well-designed biomimetic topographical cues can trigger the integrin-mediated cell adhesion pathway and provide an unparalleled opportunity for chondrogenic differentiation.

In addition to topography, surface chemistry also plays a critical role in cell adhesion, proliferation, and morphology. Surface chemistry can be effectively used to control the phenotype and promote the expression of lineage-specific markers in differentiated MSCs [154]. The desired result can be usually obtained by selective modulation of the proper combination of surface topography and chemistry [155]. The impact of surface topography on cell behavior strongly depends on the surface chemistry and is highly context specific. For instance, it has been reported that the surface chemistry was the main effective parameter on the regulation of cell adhesion on β-tricalcium phosphate surfaces whereas, for HA, surface topography was the main factor in the promotion of cell proliferation or differentiation [156]. The interplay between surface chemistry and surface topography was well investigated by Nemeth et al. to enhance chondrogenesis in DPSCs [157]. In the study, a nanopatterned photocurable hydrogel containing PEGDMA, GelMA, and HA was prepared by capillary force lithography. Incorporating GelMA supported cell adhesion and adding HA also enhanced the in vitro chondrogenic differentiation of DPSCs in the PEGDMA hydrogel through the formation of spheroids [157]. The expression of chondrogenic markers significantly increased in DPSCs cultured on nanopatterned PEG–GelMA–HA scaffolds compared with the cells cultured on tissue culture polystyrene and the unpatterned PEG–GelMA–HA scaffolds [157]. These findings showed that the nanoscale topography alone did not significantly improve the chondrogenic differentiation of DPSCs. However, the combination of nanotopographical cues and chemical modification of hydrogel with HA significantly changed the chondrogenic markers [157].

#### 3.4 Effect of surface stiffness on stem cell chondrogenesis

In addition to the effect of topography on cell adhesion, morphology, and differentiation, surface stiffness also regulates stem cell behavior (Table 2). Surface stiffness is a physical characteristic, which predominantly arises from the substrate chemistry and controls stem cell lineage differentiation. There is no widely accepted value for the stiffness modulus to specifically determine the fate of stem cells. Also, there is no relation between the stiffness value of native tissue in which these cells reside and synthetic materials, which would induce lineage differentiation. To study the effect of surface stiffness on MSC differentiation into osteoblasts and chondrocytes (two closely related cell phenotypes), different hydrogels with elastic moduli ranging from 0.1 to 310 MPa were prepared by varying monomer concentrations [173]. The results showed that both osteogenic and chondrogenic differentiations occurred on substrates with a softer surface. However, the effect of softer substrates on committed osteoblasts was entirely different from committed chondrocytes [173].

Naturally occurring stem cell differentiation is the final product of the combined effect of chemical and physical cues as well as cell–cell interactions in the context of the ECM [234]. Therefore, the integration of different types of ECM mimetic cues appears to be beneficial for a differentiation program. For example, the expression of ALP, which is an early protein marker for osteogenesis, was promoted by the interplay of substrate stiffness and cell–cell contact [235]. However, this interplay was not observed in the induction of late osteogenesis upon analysis by calcium deposition.

In an attempt to elucidate the interplay between mechanical cues and surface topography, researchers conducted an exhaustive study to investigate their combined effect on the chondrogenic differentiation of MSCs [234]. Three polymeric films, PCL, PLA and polyglycolide (PGA) with different mechanical stiffness (PCL < PLA < PGA) were imprinted by nanolithography to produce nanopillar and nanograting patterns to culture MSCs. The results showed that the proliferation rate of MSCs significantly increased in the polymers with the nanograting pattern compared to those with a nanopillar pattern, regardless of the surface stiffness. Therefore, topography significantly influenced the growth rate of MSCs in the chondrogenic medium, apart from the stiffness of the material [234]. Surprisingly, the results of chondrogenic differentiation analysis differed in polymers with the same stiffness and different topography and vice versa. Overall, chondrogenic differentiation was superior on the softer substrate compared to the stiffer substrate and enhanced on the nanopillar topography compared to the nanograting pattern [234]. The combination of the softer surfaces with nanopillar topography generated hyaline-like cartilage. Nanograting resulted in superficial/fibro zone-like cartilage. The nanopillar-patterned stiff surface increased the expression of hyaline/fibro/hypertrophic cartilage while the stiff polymer with the nanograting topography did not significantly induce the chondrogenic phenotype [234]. Overall, these results have shown that cell behavior is regulated by a combination of several parameters, which may elicit a completely different phenotype compared with either cue alone.

Although the mechanisms by which cells can sense and respond to mechanical cues are not fully understood, the details of the mechanotransduction pathway have recently been investigated; especially in the context of chondrocyte biology. The results of the study of the effect of surface stiffness on the chondrocyte phenotype showed round cell morphology and proper cytoskeleton tension on soft substrates compared to stiff substrates [174]. The RhoA/ROCK signal pathway was suggested to be responsible for the promotion of the chondrocyte phenotype on this substrate. In line with this observation, researchers proposed that a multilevel mechanotransduction pathway sensed ECM stiffness [175]. According to this model, mild stiffness would induce the ROCK pathway, which leads to the expression of autocrine TGF-β, phosphorylation of a key TGF-β effector (Smad3), and the resultant induction of the Col2α1 gene. Therefore, the substrate stiffness sensing pathway continues inside the cell via a hierarchical multilevel mechanism. The combination of surface stiffness and exogenous TGF-β also induces cells for a synergistic response such that chondrocyte cells express the chondrogenic markers more robustly than either cue alone [175]. Hence, surface stiffness can be used as a co-activation factor in differentiation programs chemically induced by growth factors [175]. For instance, matrix stiffness can exhibit a bifunctional effect of TGF-β on MSCs to differentiate into either chondrogenic lineage or smooth muscle cells (SMCs) depending on the degree of the substrate stiffness [236]. In this study, the results showed that surface stiffness was a determining factor to control the MSC fate such that differentiation into SMC lineage improved on stiff matrices while its differentiation into the chondrogenic lineage was promoted on soft matrices [236]. To better comprehend the effect of matrix elasticity on the in vitro formation of articular cartilage, researchers investigated the redifferentiation capacity of chondrocytes in three different hydrogels (fibrin, silk/fibrin, and PEG-dextran) with a broad spectrum of tissue stiffness ranging from 1 kPa up to 30 kPa [176]. In addition, these chondrocytes were stimulated using TGF-β3 to understand the potential synergistic effects of stiffness and growth factor. The results of this study clearly showed that the fibrin hydrogels with a Young’s modulus of 30 kPa, which is similar to that of the perichondral space, led to the redifferentiation of chondrocytes [176]. Chondrocytes cultured in pure fibrin hydrogels produced an ECM-enriched microenvironment, especially when stimulated with TGF-β3. The silk/fibrin hydrogel had an adverse effect on the redifferentiation capacity of chondrocytes, although it supported high cell viability [176]. Generating a functional tissue model of articular cartilage is very important in TE programs. Given that only redifferentiated chondrocytes can generate cartilage in vitro, the simultaneous use of surface stiffness and TGF-β can support the redifferentiation capacity of chondrocytes to produce functional tissue models [175–176].

## Conclusion

OA is a painful, chronic joint disease, which can lead to disability and decreased life quality in the elderly and in athletes. Although many efforts have been made to repair osteochondral defects, chronic joint diseases continue to threaten people’s health. Over the past years, regenerative medicine and TE have provided opportunities to treat degenerative diseases because of the technical progress in biomedical engineering. In this paper, recent developments in osteochondral tissue engineering were reviewed with examples from recent studies highlighting the importance of micro- and nanotechnology in tissue engineering applications. Afterward, the importance of various factors in designing proper scaffolds to regenerate cartilage tissue was discussed. Among all effective factors, biological cues, microscale/nanoscale topographical cues, and surface stiffness have received the most attention in the studies reviewed here. In the case of biological cues, it has been found that the application of such cues in scaffold structures can provide a desirable microenvironment for cell attachment, spread, migration, proliferation, and differentiation [237]. Regarding the microscale/nanoscale topographical cues, the findings demonstrate that the surface architecture impacts cell behavior and expression of chondrogenesis markers [165,215]. Hence, a scaffold with well-designed topographical cues can trigger the receptor-mediated cell adhesion pathway and provide a unique opportunity for cartilage differentiation. Stiffness can facilitate cartilage formation by regulating the ROCK pathway, which is responsible for promoting the chondrocyte phenotype [174]. However, the literature on surface architecture differs greatly in methodology, and the available data are dispersed. Therefore, designing a suitable scaffold with all the optimal attributes that meet the needs of chondrocytes is not an easy task and takes time. But we believe that with the current state of the art, it is possible to fabricate a reinforced scaffold with mechanical properties similar to those of native tissue. The growing and rapid development of microscale/nanoscale materials has offered a wide variety of novel approaches and platforms for treating osteochondral lesions. Microspheres, nanoparticles, nanofibers, nanotubes, and other micro-/nanotechnology-based drug delivery systems provide new platforms for the development of therapeutic strategies of OA. These micro- and nanostructures can also be designed to construct new multifunctional scaffolds and or incorporate them into hydrogel networks to provide a controlled release or improved mechanical characteristics. A microsphere-based controlled release strategy obviates the need for intermittent addition of growth factors and enables the in situ differentiation of MSCs [23]. The incorporation of NPs loaded with bioactive agents in hydrogels provides the scaffold with controlled release properties and improves its mechanical properties [54,70]. Electrospun nanofibers are excellent building blocks, which can be used as reinforcing agents to enhance the mechanical properties of fibrous scaffolds. Although the application of CNTs as fillers in combination with other compounds has shown encouraging properties for cartilage TE [76,127], there are ambiguous results on the CNT toxicity, many of which are related to differences in the dose used, nanotube size, purity, and routes of administration [238]. Therefore, to find out whether the inflammatory responses found by several groups in vivo outweigh the benefits of nanotubes, long-term in vivo research is needed. Despite the potential therapeutic effects of microscale/nanoscale materials, many challenges still exist in the research and application of these materials that need to be solved. The most limiting aspect of this type of study is the translation of these microscale/nanoscale materials from bench to bedside. Time and economic investment are required to assess the safety of these materials. In addition, simultaneous control of structural, biochemical, biomechanical properties, and degradation rate of scaffolds is another challenge. Meanwhile, onset and progression of OA are strongly associated with joint inflammation [17]. Hence, one of the main challenges of its clinical treatment is the regeneration of cartilage in an active inflammatory environment. Therefore, finding a safe, effective, and low-cost treatment with anti-inflammatory effects that can prevent the progression of the disease and restore the normal function of damaged cartilage is needed. Given the higher possibilities of these novel technologies, it is not improbable that one day OA can be cured. To reach this promising objective, future research should be focused on studying and evaluating the application of these microscale/nanoscale materials in large animal models to gain an improved comprehension of the feasibility of their translation from bench to bedside.
